# RACIAL VARIATION IN THE RELATIONSHIP BETWEEN LIFE-COURSE TURNING POINTS AND LIFE SATISFACTION

**DOI:** 10.1093/geroni/igz038.1634

**Published:** 2019-11-08

**Authors:** Dawn C Carr, Ben L Kail, Rocio Calvo

**Affiliations:** 1 Florida State University, Tallahassee, Florida, United States; 2 Georgia State University, Atlanta, Georgia, United States; 3 Boston College, Boston, Massachusetts, United States

## Abstract

According to many social gerontologist and life course scholars, major life course transitions, referred to as “turning points,” have a significant impact on well-being. Although the relationship between major later life turning points and general well-being is fairly well understood, it is unclear whether there is systematic racial/ethnic variation in response to turning points in general. Moreover, much of sparse research on racial/ethnic variation that does exits overlooks how Hispanic Americans may respond differently to turning points than do either white Americans or African Americans. To that end, in this paper, we draw on life course theory to assess whether the relationship between retirement and the death of a spouse (i.e. turning points) and life satisfaction (a measure of well-being) vary by race/ethnicity. We focus on differences between whites, Hispanics, and African Americans. Moreover, we draw on stress process theory to identify mechanisms that may explain any observed racial/ethnic variation in these relationships. Using the Health and Retirement Study, in preliminary results we find: 1) before adjusting for turning points, Hispanics appear to have higher life satisfaction than whites, and African Americans do not differ significantly from whites; 2) however, after adjusting for turning points, only Hispanics who make life course transitions have significantly higher life satisfaction than whites; and 3) this higher life satisfaction observed among Hispanics who experience turning points is largely not accounted for by several factors derived from stress process theory.

